# Employee Leadership Emergence and His/Her Own Innovative Behavior: Role-Based Emotional Experience as Mediator

**DOI:** 10.3390/bs15040443

**Published:** 2025-03-31

**Authors:** Tianwen Liu, Guangsheng Zhang

**Affiliations:** 1 Business School, Faculty of Economics, Liaoning University, Shenyang 110036, China; 4022210070@smail.lnu.edu.cn; 2 Department of Finance and Economics, Xuchang Vocational Technical College, Xuchang 461000, China; 3 Business School, Shenyang University, Shenyang 110044, China

**Keywords:** leadership emergence, innovative behavior, sense of power, self-efficacy

## Abstract

In the VUCA (which means volatility, uncertainty, complexity, and ambiguity) era, companies increasingly value the emergence of employee leadership as a complement to formal team leadership. Meanwhile, employee innovative behavior, as an important source of firm innovation, has gradually become a key element for the sustainable development of enterprises. Both employee leadership emergence and innovative behavior have significant impacts on the sustainable growth of the employees and companies, yet the relationship between the two has been seldom studied. Whether employee leadership emergence can promote the informal leader’s innovative behavior, thereby achieving the mutual growth of employees and enterprises, has not been tested. Against this backdrop, this study constructs a moderated mediation model from the perspective of leadership role activation to explore the relationship and underlying mechanisms between employee leadership emergence and innovative behavior. By analyzing 304 paired sample data from technology companies in Guangzhou, China, this study finds that employee leadership emergence can influence informal leader’s innovative behavior through the sense of power. Employee self-efficacy strengthens the power perceptions brought about by employee leadership emergence, thus facilitating its positive impact on innovative behavior. This study provides insights into how companies can achieve sustainable growth for both employees and enterprises through employee leadership emergence by revealing the relationship and underlying mechanisms between employee leadership emergence and the informal leader’s innovative behavior.

## 1. Introduction

In the knowledge economy era, innovation has become the key driver of sustainable and healthy corporate development (Liao et al., 2008). Unlike the industrial era, the widespread improvement in the employees’ knowledge reserves and skill levels in the knowledge society has led companies to no longer view research personnel, executives, or expert teams as the sole sources of innovation (Opland et al., 2022). Employee-driven innovation has become an important form of innovation today (Lua et al., 2024). In this context, relying solely on the existing knowledge systems and operational processes is no longer sufficient to meet the rapidly changing demands of the era. Instead, there is an increasing need for creative work from employees, in order to avoid knowledge inertia (Liao et al., 2008). This shift requires organizations to not only focus on the proficiency of the employees’ professional skills in their current roles but to enhance employee role diversity to support sustainable growth, enabling them to respond more flexibly to work challenges and to seize opportunities. Encouraging employees to demonstrate more innovative behaviors has become crucial for companies’ sustainable growth in an ever-changing business environment (Dong et al., 2017).

Existing studies often view employees as passive followers of formal leaders, finding that employee innovative behaviors are influenced by superior leadership (Lee et al., 2020), such as servant leadership (Song et al., 2024), self-sacrificial leadership (Z. Xu et al., 2022), transformational leadership (Hai et al., 2022), and empowering leadership styles (Hoang et al., 2021). However, under the context of the knowledge economy and the national strategy of innovation-driven development, leadership styles have undergone significant changes (Hanna et al., 2021). The traditional top-down formal leadership model in organizational structures is no longer effective in responding to the rapidly changing external business environment. Companies are increasingly focusing on informal leadership provided through active participation by team members (Hao et al., 2017). This phenomenon, where a team member, without formal authorization, is recognized and followed by other members due to their leadership qualities, thus influencing the team, is defined as employee leadership emergence (Badura et al., 2022; Hanna et al., 2021). The greater the potential leader’s influence on the team, the higher the degree of leadership emergence (Hanna et al., 2021).

Unlike the binary relationship between formal leaders and employees, employees who demonstrate leadership emergence simultaneously play the dual role of informal leader and employee, with both roles embodied in one individual. Due to this unique leadership emergence, the existing studies have found that employees who present leadership emergence can promote team performance (Zhang et al., 2012), as well as their own performance, such as individual work performance (Zhang et al., 2012). However, current research on the impact of employee leadership emergence on the informal leader him/herself mainly revolves around distal outcomes, such as individual work performance, with limited attention given to proximal outcomes like innovative behavior (Hao et al., 2017). Additionally, there is a lack of research on the internal mechanisms of leadership emergence’s effects, making it still an unexplored “black box” (Badura et al., 2022; Hanna et al., 2021).

Research has pointed out that leadership role identity greatly influences the leader’s behavioral performance (Gjerde & Ladegård, 2019). Role identity can be understood as a series of meanings associated with the role or position an individual occupies in an organizational environment (Stets & Serpe, 2013). For instance, a leadership role identity might include meanings such as “responsible for directing the team” and “planning action plans”. Role identity helps individuals understand the role they are expected to play in the organizational social environment (Turner, 2006), and by providing positive emotional experiences, it drives role enactment, influencing self-perception and behavioral performance (Stets & Burke, 2000). For employees who exhibit leadership emergence, even though their leadership role identity is not formally authorized by the organization, the emotional recognition of their leadership traits by other team members can still bring them a sense of power and a positive emotional experience (Anderson et al., 2012). Therefore, we hypothesize that employee leadership emergence can influence their innovative behavior by encouraging them to think from the perspective of a leadership role identity, through the sense of power they experience in interactions with followers. In other words, we believe that the emotional experiences in role enactment may serve as the key to unlocking the internal mechanism of leadership emergence’s effects.

Based on the conclusions of this study, we make the following contributions to the existing literature. First, the existing research on the antecedents of employee innovative behavior has mainly focused on the top-down influence path of formal leadership (Lee et al., 2020; Song et al., 2024; Z. Xu et al., 2022; Hai et al., 2022; Hoang et al., 2021), with little research on the bottom-up influence path of employee leadership emergence. This study fills this gap and points out that employees who exhibit leadership emergence are both the source of leadership and the practitioners of innovative behavior. Therefore, employee leadership emergence affects their own innovative behavior through the positive emotional experiences during the leadership role enactment process. This conclusion enriches the research on the antecedents of employee innovative behavior. Second, the current studies about leadership emergence have mainly focused on the antecedents and ignored the potential consequences of it. The actual impacts leadership emergence can have on both employees and organizations are seldom touched. What is even more unfortunate is that the existing limited literature on the effects of leadership emergence only discusses its direct impact, without further exploring its underlying mechanisms (Badura et al., 2022; Hanna et al., 2021). Third, we believe that conducting this study in the Chinese context is of significant importance. China’s rapid economic development is closely linked to the globalization process, which provides a unique perspective for observing leadership emergence and innovative behavior. Traditional Chinese culture places more emphasis on collectivism and often exhibits a higher level of team power distance (Hamamura et al., 2021), which could become an obstacle to the employees’ identification with informal leadership roles. Although, in the knowledge economy era, companies urgently need employees to actively participate in innovation as informal leaders. It is of practical significance to explore whether the sense of power based on leadership role identity can act as a mediating variable between the employees’ leadership emergence and their innovative behavior, given the Chinese cultural context.

In summary, this study analyzes the black box of the relationship between the employee leadership emergence and innovative behavior from the perspective of the role identity emotional experience. Table 1 compares the contributions of this study with existing research.

## 2. Theory and Hypotheses

### 2.1. Theoretical Background

#### 2.1.1. Social Exchange Theory

Social exchange theory (SET) is a sociological and psychological theoretical framework used to understand interpersonal relationships. SET suggests that human behavior can be understood as an exchange process, where most behavioral decisions in social interactions are based on the comparison of benefits and costs. Driven by the pursuit of different values, individuals make behavioral decisions based on past experiences and current situations (Homans, 1961), meaning individuals weigh their inputs against the rewards to decide whether to continue a particular relationship (Blau, 1964). Traditional research on employee innovation often focuses on the exchange relationships between employees and formal leaders. However, as more and more companies encourage the emergence of informal leadership within teams, social exchange can be further extended to include the relationship between emergent leaders and followers.

#### 2.1.2. Identity Theory

Identity theory (IT) originated in the fields of sociology and social psychology in the mid-20th century, building on the foundation of symbolic interactionism (Stryker, 1980). First proposed by Sheldon Stryker in 1968 (Stryker & Burke, 2000), the theory emphasizes the influence of social structures on individual identity and explores how individuals construct a self-concept through role enactment in social interactions.

The core concepts of identity theory are “role” and “identity”. Within social structures, a role is defined as the behavioral expectations assigned to a specific position (Burke & Stets, 2009). Individuals hold varying perceptions of their own traits—for example, “I think I am a sociable person”. This self-recognition of personal traits is termed person identity. When such a person identity becomes embedded in a social relationship and receives positive interaction from others, it evolves into role identity (Stets & Serpe, 2013), such as “I am the informal leader within the team”. The activation of role identity depends on the breadth and depth of social relationships (Stets & Burke, 2000), rather than their formality. Each role carries societal meanings and expectations, which individuals internalize to shape their identity. While similar to self-categorization in social identity theory, identity theory places greater emphasis on the direct influence of roles on individual behavior.

### 2.2. Leadership Emergence and Employee Innovative Behavior

Leadership emergence is characterized by the phenomenon where potential leaders within a team display leadership traits and are recognized and followed by potential followers (Badura et al., 2022). Some scholars define it as the phenomenon where a member of the team influences the team without formal authorization (Hanna et al., 2021). It is clear that leadership emergence is a product of the interaction process between potential leaders and potential followers. Therefore, we choose to explain the relationship between employee leadership emergence and their innovative behavior through social exchange theory (SET). When an employee is recognized by other colleagues and influences the team, we say that the employee exhibits leadership emergence. As an exchange for the colleagues’ recognition, informal leaders become more proactive in learning and adapting to their leadership roles and exhibit more proactive work behaviors to drive team development, meeting the followers’ expectations (Schyns et al., 2020). Employee innovative behavior is a typical example of proactive behavior that benefits the team (Parker & Collins, 2010). Furthermore, as a positive response to supporting followers, employees who demonstrate leadership emergence and exhibit more internalized work motivation are more likely to engage in innovative behavior (Amabile & Pillemer, 2012). This, in turn, leads followers to offer more recognition and support to the informal leader, further promoting their positive feedback. In this process of reciprocal needs exchange, the innovative behavior of employees demonstrating leadership emergence is continuously reinforced.

Therefore, we propose the following hypothesis:

**H1:**  *There is a positive relationship between employee leadership emergence and their innovative behavior.*

### 2.3. The Mediating Role of Power Perception

According to identity theory, when an individual is embedded within a social structure and plays a certain role due to their personal identity, they are assigned a corresponding role identity by that social role (Stets & Serpe, 2013). For potential leaders with leadership traits, these traits represent their personal identity (e.g., a confident and charismatic individual). When the leadership traits of a potential leader are recognized by other team members, a social relationship between the informal leader and the followers is formed. At the same time, we say that the potential leader has exhibited leadership emergence. In this social relationship, the individual demonstrating leadership emergence plays the role of a leader without formal authorization. According to identity theory, between the employee identity and the leadership role identity, employees tend to adopt the leadership role identity that brings more positive emotional experiences (Davis et al., 2019). Furthermore, the positive emotions experienced when an individual is in a social relationship and playing a role will affect their identity perception and behavioral performance (Stets & Burke, 2000). Therefore, this study seeks to uncover the internal mechanism between employee leadership emergence and innovative behavior from the perspective of emotional experiences in the employee leadership role enactment process.

Power perception refers to an individual’s perception of their ability to influence others or other groups (Anderson et al., 2012). Power perception is often closely related to an individual’s status in a social structure. Individuals in higher positions tend to experience a stronger power perception due to having more resources, information, or social support (Anderson et al., 2012). Previous studies have shown that an employee’s status in an organization can stem from both formal authorization and informal recognition by other members (Agneessens & Wittek, 2012). As Leavitt (2004) states, a leader does not need to give orders to be perceived as powerful; they only need to be able to influence the behavior of others (Leavitt, 2004). Therefore, for employees exhibiting leadership emergence, even though their leadership identity has not been formally authorized, the informal recognition of their leadership traits by team members will still enhance their power perception.

At the same time, according to the approach–inhibition theory of power, power perception influences people’s choices between the approach system and the inhibition system (Anderson & Berdahl, 2002). It is generally believed that the approach system regulates behavior related to rewards and helps individuals overcome difficulties in pursuit of rewards (Higgins, 1998). By contrast, the inhibition system is thought to regulate behavior related to punishment, causing individuals to experience anxiety and actively avoid behaviors that may lead to discomfort or punishment when they sense potential threats (Higgins, 1998). Therefore, when an employee experiences high power perception, due to having more resources, information, and social support, their behavior is influenced by the approach system (Anderson & Berdahl, 2002). At this point, the individual will be more eager to acquire new knowledge and actively face challenges, showing more innovative behavior (Gervais et al., 2013). When the individual perceives low power, they are often on the margins of social relationships and face more social or material threats (Anderson & Berdahl, 2002). In this case, their behavior, influenced by the inhibition system, will avoid risky innovative behaviors (Gervais et al., 2013) to avoid failure.

In conclusion, when employee leadership emergence is higher, emotional support from colleagues enhances their power perception. This positive emotional experience further influences their behavioral performance. Specifically, the behavior approach system activated by high power perception encourages more proactive innovative behavior.

Thus, we propose the following hypotheses:

**H2a:**  *There is a positive relationship between employee leadership emergence and power perception.*

**H2b:**  *There is a positive relationship between power perception and employee innovative behavior.*

**H2c:**  *Power perception mediates the indirect relationship between employee leadership emergence and employee innovative behavior.*

### 2.4. The Moderating Role of Self-Efficacy

Previous research has pointed out that identity recognition related to roles is primarily driven by the internal motivation of self-efficacy (Stets & Burke, 2000). Self-efficacy is defined by Bandura (1978) as an individual’s subjective belief system about their ability to perform specific roles, effectively complete tasks, or successfully cope with environmental challenges. This concept holds special significance in the field of organizational behavior: compared to simple skill assessments, self-efficacy places more emphasis on an individual’s cognitive judgment of their dynamic boundaries of ability (Bandura, 1978). This judgment directly affects their degree of role involvement and behavioral choice strategies.

In terms of emotional experience, individuals with high self-efficacy tend to exhibit significant positive emotional advantages during role enactment. The identity theory model outlined by Stets and Burke (2000) reveals that, when an individual’s actual role performance aligns with their internal self-schema, it triggers a positive emotional feedback mechanism. In organizational contexts, employees with high self-efficacy typically hold more positive self-expectations (Bandura, 1978). This expectation influences their emotional feedback through the “anticipatory self-appraisal” mechanism—when they exhibit leadership emergence within a team (such as actively participating in task decision-making and gaining recognition from team members), it forms a reinforced cognitive match between their abilities and roles (DeRue & Ashford, 2010). This cognitive alignment not only strengthens their sense of confirmation in the leadership role identity but, more importantly, activates “perceived influence efficacy”, which is the individual’s evaluation of their ability to change others’ attitudes, behaviors, or team outcomes (Anderson et al., 2012). Traditional research emphasizes structural power that stems from controlling key resources (French & Raven, 1959), while emerging psychological perspectives reveal that experiential power is equally important—it arises from an individual’s subjective perception of their social influence (Anderson & Galinsky, 2006). Based on this, this study proposes that, when individuals with high self-efficacy exhibit leadership emergence behaviors, their intrinsic “belief in influencing others’ efficacy” will catalyze an increase in power perception.

Thus, we propose the following hypothesis:

**H3a:**  *Self-efficacy positively moderates the relationship between employee leadership emergence and power perception.*

Furthermore, this study suggests that self-efficacy not only moderates the relationship between employee leadership emergence and power perception but moderates the indirect relationship between employee leadership emergence and their innovative behavior. Specifically, employees with higher self-efficacy will experience greater power perception after exhibiting leadership emergence. Subsequently, higher power perception will make it easier for employees to be influenced by the approach system of power, leading them to exhibit more innovative behaviors that challenge the status quo.

Thus, we propose the following hypothesis:

**H3b:**  *Self-efficacy positively moderates the indirect relationship between employee leadership emergence and employee innovative behavior through power perception. That is, the higher the self-efficacy, the stronger the effect of employee leadership emergence on innovative behavior through power perception.*

**Figure 1:**
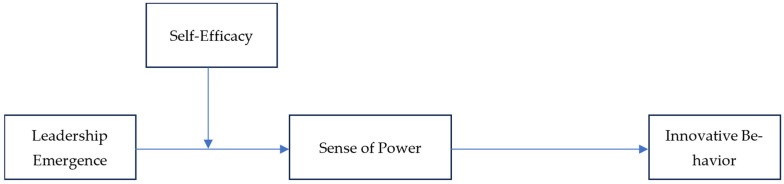
Conceptual Model.

Our conceptual model is shown in Figure 1.

## 3. Research Design

### 3.1. Research Sample and Data Collection

This study selected 12 high-tech companies from multiple cities in Guangdong Province as the subjects for the questionnaire distribution, covering industries such as software development, biotechnology, and game development. This study selected high-tech companies as the research sample for two key reasons. First, existing research has demonstrated that organizations characterized by flat structures, diverse teams, and knowledge-intensive environments are conducive to informal leadership emergence among employees (Hsu et al., 2017). Second, compared to traditional industries, employee innovative behavior is more prevalent in technology-driven firms. Following the existing research, we tested the relationship between employee leadership emergence and innovative behavior within high-tech companies (Hsu et al., 2017).

To minimize the impact of common method bias on the relationships between variables, this study employed anonymous responses and matched superior–subordinate pairs, collecting data over two time periods with a 20-day interval between them. To improve the authenticity and accuracy of the questionnaire data, the researchers communicated with the management of the surveyed companies about the study’s objectives and the anonymous nature of the survey before distributing the questionnaires. The data collection was conducted using electronic questionnaires.

In the first phase (Time 1), the employee (who is not a formal leader in the team) part questionnaire included personal information, while the supervisor (the direct supervisor of each employee) part questionnaire focused on employee leadership emergence. This stage involved communication with each company’s human resource department to decide a sample size based on the company’s size, to ensure that each company has the similar proportion of samples selected. Employees were randomly chosen from each company to fill out the questionnaire by scanning a QR code. A total of 365 paired responses were received in this phase.

In the second phase (Time 2), the employee part questionnaire included power perception, self-efficacy, and employee innovative behavior. After receiving the questionnaires, those with regular patterns of responses or serious data missing were excluded. Ultimately, 304 valid leader–employee pairing questionnaires were retained, resulting in an effective recovery rate of 73%. In the final sample, 54.3% of respondents were male, and 45.7% were female; the average age was 28.41 years (SD = 3.48); the average work experience was 3.95 years (SD = 2.33); 80.1% of employees had a bachelor’s degree or higher.

### 3.2. Variable Measurement

The scales used in this study are based on established, reliable scales from both domestic and international sources. To ensure the quality of the measurement scales, English-language scales were translated and back-translated, incorporating adjustments based on the relevant literature and the context of this study. The final versions of the scales were reviewed by HR experts from the data source companies, who provided feedback and suggestions. After multiple rounds of communication, the final contents of the scales were confirmed. All scales were evaluated using a Likert 5-point scale, where 1 represents “strongly disagree” and 5 represents “strongly agree”.

Leadership Emergence (LE): The measurement of leadership emergence is based on a 3-item scale developed by Marinova et al. (2013) and was evaluated by the leaders. A typical item is, “This employee is becoming an effective leader in the team.” Previous research has shown that scoring, as a measurement method, has good psychometric properties (Taggar et al., 1999) and effectively measures employee leadership emergence (Marinova et al., 2013; Taggar et al., 1999). The internal consistency coefficient (Cronbach’s α) of this scale was 0.80.

Power Perception (SP): The measurement of power perception is based on an 8-item scale developed by Anderson and Galinsky (2006) and was filled out by employees to reflect their perceptions of having power in their relationships with other team members. A typical item is, “I can make others listen to what I say.” Consistent with the previous research, this scale shows high internal consistency, with a Cronbach’s α of 0.92.

Employee Innovative Behavior (IB): Employee innovative behavior was self-reported by employees. The specific measurement used is a 6-item scale developed by Scott and Bruce (1994), which is widely used to measure employee innovative behavior. A typical item is, “I often explore new work techniques and methods in my job.” The internal consistency coefficient (Cronbach’s α) of this scale was 0.90.

Self-Efficacy (SE): Bandura (1978) pointed out that self-efficacy can be quantified by measuring an individual’s belief in their ability to successfully achieve desired outcomes through specific behaviors. Based on this, Jones (1986) developed an 8-item self-efficacy scale. A typical item is, “My past experiences and achievements make me believe that I can perform well in the current team.” The internal consistency coefficient (Cronbach’s α) of this scale was 0.93.

Control Variables: Since the research subjects in this study are individual employees, in order to ensure the generalizability of the results, we controlled for sample characteristics based on existing similar studies (Miao et al., 2023). These control variables include gender (Gen), age (Age), education level (Edu), and work experience (Ten).

## 4. Analysis of Research Results

### 4.1. Reliability and Validity Testing

In this study, confirmatory factor analysis (CFA) was conducted using Amos 24 software to test the reliability and validity of the model. The results are shown in Table 2 and Table 3. Table 2 shows that the composite reliability (CR) of the model’s expected variables is greater than 0.70, and the average variance extracted (AVE) is greater than 0.5, meeting the criteria recommended by (Hair, 2009), indicating that the scale has good internal consistency. Table 3 presents the model fit indices, comparing the four-factor measurement model, which includes employee leadership emergence, power perception, innovative behavior, and self-efficacy, with three alternative models (three-factor, two-factor, and one-factor models). It was found that the four-factor model provides a better fit to the actual data (CMIN = 468.811; df = 269; CMIN/df = 1.743; IFI = 0.959; TLI = 0.954; CFI = 0.959; RMSEA = 0.050), demonstrating good discriminant validity.

### 4.2. Common Method Bias Test

To test for potential common method bias, this study used exploratory factor analysis for the Harman one-factor test. All items from the variables were included in the exploratory factor analysis. The results showed that the first unrotated factor explained 37.36%, which is below the 40% critical threshold. Therefore, it is concluded that this study is not significantly affected by common method bias. As a supplementary test for common method bias, this study further employed the latent method factor control (ULMC) approach. The ULMC method involves adding a common method factor to the baseline model and then conducting confirmatory factor analysis. If the changes in the model’s fit indices (RMSEA and SRMR) exceed 0.05, and the changes in CFI and TLI are greater than 0.1, it would indicate serious common method bias issues (Podsakoff et al., 2003). After adding the method factor, the new five-factor model’s fit indices were (χ^2^ = 411.193; CFI = 0.967; TLI = 0.960; RMSEA = 0.046), with no significant changes. Thus, this study does not have severe common method bias issues and can proceed with further analysis.

### 4.3. Data Normality Test

The normal distribution of data is a prerequisite for ensuring the accuracy of regression results. After testing, the absolute values of skewness and kurtosis for all variables are less than two. George and Mallery (2010) suggest that when the absolute values of skewness and kurtosis are less than two, the data can be accepted as following a normal distribution.

### 4.4. Descriptive Statistics and Correlation Analysis

The means, standard deviations, and correlation coefficients of the variables in this study are shown in Table 4. As can be seen from the table, employee leadership emergence is positively correlated with power perception (r = 0.491, *p* < 0.001) and with employee innovative behavior (r = 0.514, *p* < 0.001). Power perception is positively correlated with employee innovative behavior (r = 0.597, *p* < 0.001), providing preliminary support for our hypotheses.

### 4.5. Hypothesis Testing

#### 4.5.1. Main Effects and Mediation Effect Analysis

This study used SPSS 23.0 for hierarchical regression analysis. Control variables, employee leadership emergence, and power perception were sequentially added to the regression model. The results of the hierarchical regression for the hypotheses are shown in Table 5. Model 4 shows that employee leadership emergence is positively correlated with employee innovative behavior (β = 0.499, *p* < 0.001), confirming the main effect of Hypothesis 1, which indicates that the mediation effect test can proceed. According to Model 1, employee leadership emergence is positively correlated with power perception (β = 0.483, *p* < 0.001), validating Hypothesis 2a. According to Model 5, power perception is positively correlated with employee innovative behavior (β = 0.564, *p* < 0.001), validating Hypothesis 2b. Comparing Model 4 with Model 6, when both employee leadership emergence and power perception are included, the positive relationship between employee leadership emergence and employee innovative behavior remains significant, but the regression coefficient decreased from 0.499 to 0.297, suggesting that power perception partially mediates the positive relationship between employee leadership emergence and employee innovative behavior.

To test the significance of the mediation effect of power perception, we used the bootstrap method with 5000 resamples and a 95% confidence interval via the Process plugin in SPSS 23 software.

After including power perception, the direct effect of employee leadership emergence on employee innovative behavior was 0.297, with a 95% confidence interval of [0.200, 0.394]. Meanwhile, the indirect effect of employee leadership emergence on employee innovative behavior through power perception was 0.202, with a 95% confidence interval of [0.142, 0.272], indicating that the mediation effect of power perception reached a significant level. The indirect effect accounted for 40.5%. This further validates that power perception plays a partial mediation role in the positive relationship between employee leadership emergence and employee innovative behavior, thus confirming Hypothesis H2c.

#### 4.5.2. Moderation Effect and Moderated Mediation Test

##### Moderating Effect of Self-Efficacy

**Figure 2:**
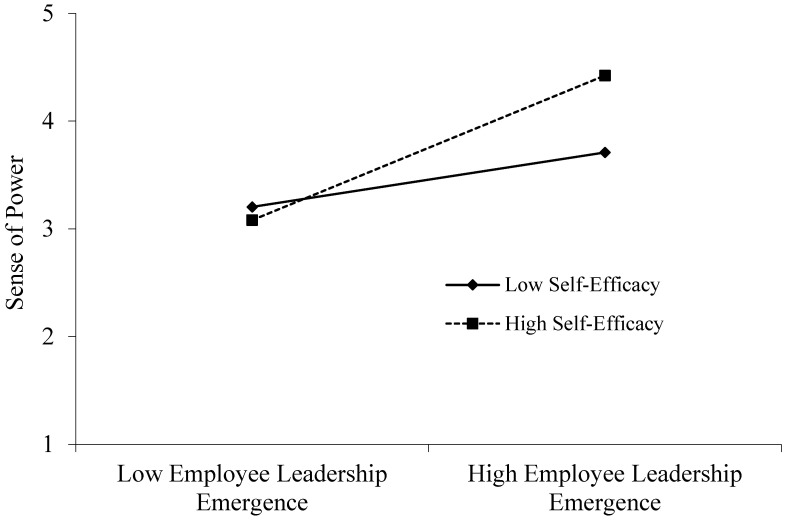
Moderating Effect of Self-Efficacy.

According to the regression results in Table 5, Model 3 shows that the interaction term between employee leadership emergence and self-efficacy significantly and positively predicts power perception (β = 0.209, *p* < 0.001). The moderating effects of self-efficacy at high levels (M + 1SD) and low levels (M − 1SD) are shown in Figure 2. Furthermore, simple slope analysis reveals that when self-efficacy is at high and low levels, the positive relationship between employee leadership emergence and power perception is more significant, with values of (β = 0.685, *p* < 0.001) and (β = 0.240, *p* < 0.005), respectively. Hypothesis 3a is supported.

##### Moderating Effect of Self-Efficacy on the Mediation Relationship

To test this first-stage moderated mediation model, this study used the Process macro Model 7 for conditional process modeling, based on 5000 bootstrap samples. The results are shown in Table 6. Table 6 reveals that, regardless of whether self-efficacy is high, medium, or low, the confidence intervals for the indirect effect do not contain 0, indicating that the indirect effect is significant. Additionally, the moderated mediation index is 0.093 (Boot 95% CI = [0.052, 0.143]), with the confidence interval not including 0. In conclusion, Hypothesis 3b is supported.

## 5. Theoretical Implications

First, this study enriches the research on leadership and employee innovative behavior. Unlike the existing studies on employee innovative behavior, which primarily focus on the vertical impact of formal leadership, this research explores the relationship between employee leadership emergence as an informal form of leadership and employee innovative behavior. This provides a new perspective for the research on leadership and employee innovative behavior. Previous studies on leadership and employee innovative behavior have often explored the relationship between formal leadership and employee innovative behavior through leadership styles (Hai et al., 2022; Song et al., 2024; Z. Xu et al., 2022) and leader–member exchange (Gao et al., 2022), among others. These studies highlight the relationship between different actors (formal leaders and employees), treating employee innovative behavior as a passive result of superior leadership influence and forming a binary research model. However, with the increasing flattening of organizational structures and the rise of employee self-management (Li et al., 2021), employee leadership emergence has gained more attention (Hanna et al., 2021). For employees who exhibit leadership emergence, they are both the source of leadership and the practitioners of innovative behavior. Thus, this study constructs a single-entity research model of leadership and employee innovative behavior by exploring informal leadership in the form of leadership emergence, analyzing the relationship between employee leadership emergence and innovative behavior from the employee’s perspective. Furthermore, the existing studies on the effects of leadership emergence are scarce (Hanna et al., 2021), and most research has focused on its direct impact on distal outcomes, such as work performance (Zhang et al., 2012), with little attention paid to proximal outcomes, such as employee innovative behavior, voice behavior, and other proactive behaviors (Hao et al., 2017). This study fills this gap.

Second, this study reveals the internal mechanism of the effects of leadership emergence from the perspective of emotional experiences during role enactment. Although a small number of studies have explored the effects of employee leadership emergence (Gerpott et al., 2019; Zhang et al., 2012), the internal mechanisms behind these effects remain an unexplored black box. Recent research has gradually focused on the impact of leadership role identity on leadership behavior (Gjerde & Ladegård, 2019). Role identity refers to the meaning attributed to an individual’s position in social interaction, which influences their emotional experiences during role enactment (Stets & Burke, 2000). Role identity can promote social interaction and influence individual behavior by providing unique emotional experiences related to that role (Turner, 2006). When employees demonstrate leadership traits and gain recognition from colleagues, a social interaction between leaders and followers is formed. At this point, employees exhibiting leadership emergence play the role of a leader. Therefore, this study finds that leadership emergence can enhance employee innovative behavior by providing employees with a sense of power. This finding offers insight into the internal mechanisms of the effects of leadership emergence.

Third, this study reveals boundary conditions of the internal mechanism in the relationship between employee leadership emergence and innovative behavior. Previous research has pointed out that emotional experiences related to role identity are influenced by self-efficacy (Stets & Burke, 2000). Since the leadership role identity formed in employee leadership emergence is not formally authorized by the organization, the extent to which employees can feel empowered by the leadership role depends on their level of self-efficacy. Employees with high self-efficacy have high confidence in their ability to perform a role, complete tasks, or meet challenges (Bandura, 1978). This study found that employees with high self-efficacy are more likely to feel empowered by the leadership role after exhibiting leadership emergence, ultimately demonstrating higher innovative behavior.

## 6. Practical Implications

First, companies should increase their attention to employee leadership emergence. This study found a positive relationship between leadership emergence and employee innovative behavior. Considering the key role of employee innovative behavior in the sustainable growth of businesses in the knowledge economy era (Lua et al., 2024), and the fact that employee leadership emergence is an intrinsic promoting factor for individual innovative behavior, employee leadership emergence should receive more attention from formal managers. Since leadership emergence often occurs in environments with minimal formal intervention (Hanna et al., 2021), companies should establish agile organizations within their businesses to provide the environmental support necessary for leadership emergence. Cross-departmental collaborative teams are a common example of agile organizational structures. For instance, each Squad in Spotify is a rapidly iterating cross-functional team with the autonomy to make decisions and design products or features. Other successful examples include Microsoft’s Azure team and Netflix’s content development team. In these agile organizational structures, employees have high autonomy, which fosters lateral leadership among employees and helps promote individual innovative behaviors.

Second, companies should provide appropriate guidance for employees who exhibit leadership emergence, as they may experience a shift in role cognition. This study, based on emotional experiences during leadership role enactment, explored the internal mechanism of the relationship between leadership emergence and employee innovative behavior. The results showed that leadership emergence enhances employee innovative behavior through increased power perception. This process involves employees transitioning from an employee role identity to a leadership role identity. Although identity theory suggests that individuals are inclined to take on more proactive role identities, playing a challenging leadership role is not easy. Traditional Chinese culture usually places more emphasis on collectivism and often exhibits a higher level of team power distance (Hamamura et al., 2021), which can be barrier for employees to treat themselves as an informal leader and achieve the sense of power in a team. For this reason, we have three suggestions. First, formal leaders should engage in communication with potential leaders to understand their psychological changes and identify their self-role positioning, providing appropriate guidance. Second, companies can host leadership training sessions for employees with potential, helping them build correct leadership role cognition. Finally, companies can organize simulated training sessions to cultivate leadership qualities in potential leaders, ensuring a smooth transition in role identity.

Third, companies should take the necessary measures to ensure that employees have high self-efficacy in their work. This study found that self-efficacy moderates the indirect relationship between leadership emergence and employee innovative behavior. Specifically, employees with high self-efficacy are more confident in their leadership role and have stronger competence motivation, which enhances the indirect relationship between leadership emergence and innovative behavior. Unlike formally authorized leaders, employees who demonstrate leadership emergence do not have their leadership role formally recognized. Therefore, for employees with low self-efficacy, even if their leadership traits are recognized by colleagues, they may not have enough confidence to feel empowered to influence others, thereby limiting their power perception. Formal leaders should provide appropriate emotional or policy support to employees with low self-efficacy, helping them gain sufficient confidence to embrace leadership roles after exhibiting leadership emergence.

## 7. Limitations and Future Research

This study explored the internal mechanism of the relationship between leadership emergence and employee innovative behavior from the perspective of emotional experiences during role enactment. Research based on a specific perspective helps us to delve deeper into the essence of the relationship but may overlook the potential influence of other relevant factors. Therefore, we acknowledge the following limitations, which can be explored in future research.

First, this study did not consider the potential role of formal leadership in the relationship between employee leadership emergence and employee innovative behavior. We explored the mechanism of leadership emergence from the perspective of employee role identity enactment and found that emotional experiences during role enactment can explain the internal logic of how leadership emergence influences employee innovative behavior. Although the rapid changes in the external environment in the knowledge economy era have forced companies to adopt agile organizational structures to meet various challenges (Silva-Martinez, 2024), and give employees more autonomy to stimulate their innovation (Liu et al., 2020), formal leadership is still an indispensable element for the sustainable and healthy growth of enterprises. Previous studies have pointed out that the lack of formal leadership can lead to conflicts and coordination issues within teams (Anderson & Willer, 2014), which reduces the contribution of informal leadership to team innovation (Dust & Ziegert, 2016). Future research should explore how formal and informal leadership can collaborate to better promote leadership emergence and employee innovative behavior.

Second, this study only conducted a boundary analysis of the internal mechanism of the relationship between leadership emergence and employee innovative behavior from an individual cognition perspective, but did not consider the potential influence of team or organizational climate. Based on the individual perspective, this study found that self-efficacy moderates the effects of leadership emergence. Hanna et al. (2021), through a literature review, pointed out that, in addition to individual characteristics, contextual factors (both internal and external to the organization) may limit the effects of leadership emergence. Internal environmental factors can include organizational culture and policies, while external factors can include market dynamics, national cultural differences (Badura et al., 2022). Furthermore, a high level of team power distance is common in Chinese companies (Hamamura et al., 2021), which can make it difficult for employees to identify the informal leader role and achieve a sense of power. Future research can construct theoretical models to explore other boundary conditions (such as guanxi and mianzi) that may influence the relationship between employee leadership emergence and his/her innovation behavior.

Third, the sample of this study was exclusively drawn from Guangdong Province, China, for two reasons. First, due to budget constraints and limitations in social network accessibility, our research resources were primarily concentrated in this province. Second, Guangdong Province hosts a large number of high-tech companies. As noted earlier, existing studies have identified R&D team members in technology firms as ideal samples for investigating informal leadership and employee innovation (Hsu et al., 2017). While this sampling strategy is justified, it may overlook other contextual characteristics, such as region-based cultural differences and organizational structure variations across enterprise types. Future studies could select more generalizable samples to further explore other intrinsic mechanisms and boundary conditions.

## 8. Conclusions

The results of this study show that there is a positive relationship between leadership emergence and employee innovative behavior. Power perception mediates this relationship. This suggests that even in a cultural context like China, where team power distance is generally high, the emergence of informal leadership can still lead to a sense of power for employees and encourage their more active participation in organizational innovation. It is evident that leadership emergence could serve as a powerful tool for organizations to unlock the employees’ potential. Furthermore, for employees with high self-efficacy, leadership emergence enhances their power perception, which in turn leads to higher innovative behavior. This indicates that employees with high self-efficacy tend to have stronger confidence in their abilities and are more willing to break conventions in order to realize their self-worth. In conclusion, this study reveals the internal mechanism of the relationship between leadership emergence and employee innovative behavior from the perspective of emotional experiences during role enactment.

## Figures and Tables

**Table 1:** Comparison with the Existing Literature.

Related Studies	Informal Leadership as Predictor	Mechanisms Exploration	Boundary Conditions	Employee Innovation as Outcome
(Lee et al., 2020; Song et al., 2024; Z. Xu et al., 2022; Hai et al., 2022; Hoang et al., 2021)	No, formal leadership as Predictor.	Yes	Yes	Yes
(Zhang et al., 2012)	Yes	No	No	No
(Chiu et al., 2021)	Yes	Yes	Yes	No
(N. Xu, 2018)	Yes	No	No	Yes
This Study	**Yes**	**Yes**	**Yes**	**Yes**

**Table 2:** Confirmatory Factor Analysis Results.

Items	Factor Loadings	SMC	CR	AVE
Employee Leadership Emergence (Marinova et al., 2013)				
LE1	0.818	0.669	0.803	0.576
LE2	0.730	0.533		
LE3	0.726	0.527		
Sense of Power (Anderson & Galinsky, 2006)				
SP1	0.773	0.598	0.920	0.590
SP2	0.768	0.590		
SP3	0.739	0.546		
SP4	0.757	0.573		
SP5	0.778	0.605		
SP6	0.776	0.602		
SP7	0.754	0.569		
SP8	0.8	0.640		
Employee Innovative Behavior (Scott & Bruce, 1994)				
IB1	0.817	0.667	0.905	0.613
IB2	0.738	0.545		
IB3	0.764	0.584		
IB4	0.752	0.566		
IB5	0.809	0.654		
IB6	0.814	0.663		
Self-Efficacy (Jones, 1986)				
SE1	0.838	0.702	0.935	0.644
SE2	0.775	0.601		
SE3	0.801	0.642		
SE4	0.763	0.582		
SE5	0.826	0.682		
SE6	0.868	0.753		
SE7	0.757	0.573		
SE8	0.786	0.618		

Notes: N = 304.

**Table 3:** Model Fit Comparison.

Fit Indices	χ^2^	DF	χ^2^/DF	RMSEA	IFI	TLI	CFI	Δχ^2^	ΔDF
4 Factors (LE, SP, IB, SE)	468.811	269	1.743	0.050	0.959	0.954	0.959		
3 Factors (LE + SE, SP, IB)	829.604	272	3.050	0.082	0.886	0.873	0.885	360.793	3
2 Factors (LE + SE + SP, IB)	2153.896	274	7.861	0.15	0.615	0.576	0.613	1324.292	2
1 Factors (LE + SP + IB + SE)	2606.362	275	9.478	0.167	0.523	0.476	0.52	452.466	1

**Table 4:** Descriptive Statistics and Interrelationships between Variables.

	Mean	SD	1	2	3	4	5	6	7	8
Gen (1)	0.543	0.499	1							
Age (2)	28.414	3.475	0.022	1						
Ten (3)	3.954	2.327	−0.018	0.781 ***	1					
Edu (4)	3.188	0.813	0.041	0.704 ***	0.399 ***	1				
EL (5)	3.594	0.882	−0.005	0.102	0.049	0.071	1			
SE (6)	3.349	1.081	0.055	0.011	0.022	0.094	0.240 ***	1		
SP (7)	3.605	0.931	0.051	0.128 *	0.057	0.174 **	0.491 ***	0.279 ***	1	
IB (8)	3.768	0.892	0.101	0.205 ***	0.104	0.264 ***	0.514 ***	0.257 ***	0.597 ***	1

Notes: (1) * *p* < 0.05 ** *p* < 0.01 *** *p* < 0.001; (2) Gen: 1 = Male, 0 = Female; Edu: 1 = High school and below, 2 = Associate Degree, 3 = Bachelor’s degree, 4 = Master’s degree, 5 = Doctoral degree.

**Table 5:** Main Effects and Mediation Analysis in Regression.

	SP			IB		
	Model 1	Model 2	Model 3	Model 4	Model 5	Model 6
Gen	0.047	0.038	0.029	0.095 *	0.066	0.075
Age	−0.034	0.009	−0.007	−0.006	0.057	0.008
Ten	−0.004	−0.026	0.008	−0.006	−0.024	−0.004
Edu	0.163 *	0.130	0.125	0.231 **	0.132	0.163 *
EL	0.483 ***	0.444 **	0.462 ***	0.499 ***		0.297 **
SP					0.564 **	0.418 **
SE		0.159 **	0.148 **			
EL × SE			0.209 **			
R^2^	0.263	0.286	0.329	0.325	0.387	0.454
Adjusted R^2^	0.251	0.272	0.313	0.314	0.377	0.443
F Value	21.301 ***	19.863 ***	20.741 ***	28.676 ***	37.677 ***	41.085 ***

Notes: * *p* < 0.05 ** *p* < 0.01 *** *p* < 0.001.

**Table 6:** Results of Moderated Mediation Analysis.

Employee Leadership Emergence → Power Perception → Employee Innovative Behavior					
Conditional Indirect Effect	Conditions	Effect	BootSE	BootLLCI	BootULCI
	Low Self-Efficacy (M − 1SD)	0.100	0.034	0.038	0.168
	Average Self-Efficacy (M)	0.193	0.032	0.137	0.261
	High Self-Efficacy (M + 1SD)	0.286	0.044	0.206	0.380
Moderated Mediation	Index	Index	BootSE	BootLLCI	BootULCI
		0.093	0.023	0.052	0.143

## Data Availability

The data that support the findings of this study are available on request from the corresponding author.
